# Suicidology Post Graduate Curriculum: Priority Topics and Delivery Mechanisms for Suicide Prevention Education

**DOI:** 10.3390/ijerph18189926

**Published:** 2021-09-21

**Authors:** Jacinta Hawgood, Karolina Krysinska, Maddeline Mooney, Ingrid Ozols, Karl Andriessen, Carmen Betterridge, Diego De Leo, Kairi Kõlves

**Affiliations:** 1 World Health Organization Collaborating Centre for Research and Training in Suicide Prevention, Australian Institute for Suicide Research and Prevention, Griffith University, Brisbane 4111, Australia; maddeline.mooney@gmail.com (M.M.); d.deleo@griffith.edu.au (D.D.L.); k.kolves@griffith.edu.au (K.K.); 2 Centre for Mental Health, Melbourne School of Population and Global Health, The University of Melbourne, Melbourne 3010, Australia; karolina.krysinska@unimelb.edu.au (K.K.); karl.andriessen@unimelb.edu.au (K.A.); 3 Department of Psychiatry, The University of Melbourne, Melbourne 3002, Australia; ingrid@mhatwork.com.au; 4 Monash Health, Faculty of Medicine, Nursing and Health Sciences, Monash University, Melbourne 3800, Australia; 5 Suicide Risk Assessment Australia, Sydney 2000, Australia; carmen@suicideriskassessment.com.au

**Keywords:** suicidology, postgraduate education, suicide prevention, suicide prevention training, curriculum, curriculum mapping, stakeholder survey

## Abstract

Background: There has been limited attention to the development and delivery of tertiary suicide prevention curricula. The aim of this work was to describe the status of postgraduate suicide prevention education, with specific attention on examining the needs of the suicide prevention sector in Australia. Method: An online survey was completed by 76 stakeholders in Australia. Current curriculum learning outcomes from Griffith University’s postgraduate suicidology programs guided the development of the survey. Results: Four key learning domains were rated highest in importance by stakeholders. According to most stakeholders, skills-based qualifications were the most relevant type of qualification, and online modulized education was the most preferred delivery mode. Half of stakeholders supported suicide prevention professional development through a combination of financial support and study leave. Conclusions: The survey provided invaluable feedback regarding the priorities of Australia’s suicide prevention sector for content domains and delivery mechanisms for tertiary suicidology education. The findings showed the preferred type of organizational (employer) support that may be provided for employees to undertake such education. These findings will inform the future developments of Griffith University’s suicidology programs and may motivate other universities to consider offering same or a similar type of education to support the suicide prevention sector toward saving lives.

## 1. Introduction

Suicide remains an important public health problem globally and in Australia. In 2019, over 700,000 people died by suicide worldwide (a global age-standardized suicide rate of 9.0 per 100,000 population) and for every suicide, there were approximately 20 people who made a suicide attempt [1]. Furthermore, with the rise of mental health impacts from the COVID-19 pandemic globally, there has been speculation that suicide attempts and suicide rates will increase [2,3]. While ongoing surveillance is of course required, to date, there is no evidence of this trend [4]. Nevertheless, critical attention should be paid to the longer-term effects of the COVID-19 pandemic on vulnerable groups, the economy, and the general population [5]. In Australia, over 3300 people died by suicide in 2019 (12.9 per 100,000 population) [6] and there were more than 28,600 hospitalizations due to intentional self-harm in Australia in 2019–2020 [7]. 

According to the WHO, suicide prevention is a global public health imperative and member countries have been called to “develop or strengthen comprehensive suicide prevention strategies in a multisectoral public health approach” [8] (p. 3). A comprehensive national suicide prevention strategy involves suicide prevention training and education, including sharing the collective voice of people with lived experience, to translate research findings into practice and to inform and build a more capable and sustainable suicide prevention sector [9,10]. In Australia, the National Suicide Prevention Strategy emphasizes promotion, prevention, and early intervention, and training and education are among the 11 elements of suicide prevention emphasized in the Fifth National Mental Health and Suicide Prevention Plan [11]. 

There is a tendency in the suicide prevention sector, and the literature, to include all types of education under the same umbrella of “suicide prevention training.” Suicide prevention education and training can be delivered in many formats, including gatekeeper training, specialized professional training, and curriculum-based tertiary education. Interchangeable use of these terms can lead to misunderstandings and misinformation at a policy, planning, and funding level, and could result in gaps in the capabilities of the suicide prevention sector. This article aimed to (1) provide an overview of the evolution and current status of suicide prevention education with a specific focus on enhancing tertiary education and (2) examine suicide prevention stakeholder views about suicidology priority curriculum content and delivery modes in Australia.

## 2. Overview 

### 2.1. Review of the Need for Suicide Prevention Education

There has been an ongoing need for enhanced training and education across the diverse stakeholder groups engaged in suicide prevention. Pompili et al. [12] called for an integration of suicidology with general medicine and observed that suicide prevention training should be “an essential component of general medical education and clinical training” (p. 128). In an Australian study, Hawgood et al. [13] found no standardized approach to suicide prevention education in medical schools, although both undergraduate medical students and general practitioners (GPs) strongly supported the introduction of a national suicide prevention curriculum. Other studies have reported widespread exposure to patients at risk of suicide among health professionals, including psychiatrists, psychiatric nurses, GPs, pediatric residents, medical staff working in emergency departments, and medical students and, therefore, a need for suicide-related education [14,15,16,17]. Similarly, although some psychology graduate training programs reportedly include a lecture (or part thereof) on suicide intervention [18], it is unclear whether the knowledge gained results in skill acquisition aligned with Cramer et al.’s [19,20] competency standards. Despite the need, adequate suicide risk assessment and intervention education appears lacking [13]. Although, there is evidence of suicide prevention education pertaining to clinical competencies emerging in tertiary programs, particularly in the U.S. [19,20]. 

A dearth of adequate training at the graduate and postgraduate levels has been reported in the field of social work [21,22,23,24] and mental health services [25,26,27]. For instance, only 21% of social workers in the U.S. received formal suicide intervention and prevention training during their Master’s-level program training, whereas 75% considered such training to be “important” or “very important” [22]. While 67% of behavioral healthcare professionals participating in the Zero Suicide Workforce Survey [27] reported that they received some type of suicide prevention training, there was a disparity between professional groups regarding the training received (e.g., 91% crisis services professional staff and 25% of adjunct therapists). At the same time, approximately 40% of behavioral healthcare professionals expressed an interest in specific suicide prevention training, such as identifying risk factors and warning signs, screening and assessment, or management and safety planning [27]. 

### 2.2. Type of Suicide Prevention Education

#### 2.2.1. Gatekeeper Training

Gatekeeper training focuses on the training of “gatekeepers,” i.e., individuals “strategically positioned to recognize a person in crisis, identify behavioral warning signs of suicide, refer a person to help, and perform any other additional capabilities that may help distressed individuals” [28] (p. 1). Gatekeepers may be community members (such as teachers, clergy members, and co-workers), as well as professionals (such as health professionals), who may encounter individuals at risk of suicide through their professional role [29]. In general, gatekeeper training increases knowledge, skills, self-efficacy, and attitudes toward suicide prevention [30,31,32,33]; however, these effects tend to decrease over time [34,35]. Furthermore, gatekeeper training is often delivered as a component of a broader multicomponent suicide prevention program, which makes it difficult to ascertain the specific effects of such training on suicidal behavior and ideation [36,37,38].

#### 2.2.2. Specialized Professional Training

There is some evidence of the effectiveness of specialized training on evidence-based suicide prevention strategies, such as suicide risk assessment, safety planning, and clinical interventions, which have been delivered to mental health professionals [39,40,41,42], school psychologists [43], medical, nursing, and pharmacy students [44,45,46], psychology students [26], social work students [23,47], and students in other health professions [40]. For instance, in Australia, first-year medical, paramedical, and pharmacy students who completed an experiential suicide awareness and intervention program reported improved skills, knowledge, and attitudes toward assessment and management of at-risk individuals [32]. Active-duty U.S. Air Force mental health professionals who attended an empirical-based workshop training reported increased confidence in assessing suicide risk and management of suicidal patients, as well as enhanced suicide care practices and clinic policy [41].

#### 2.2.3. Curriculum-Based Tertiary Education

As Muehlenkamp and Thoen [48] observed, “most of the professional training and education is delivered in piecemeal fragments, provided through brief workshops, webinars, or one-class didactic seminars” (p. 1574). Additionally, neither gatekeeper training nor suicide-specific competency-based clinical training (e.g., suicide risk assessment; see [19,20,41]) address broadly suicide prevention research, policy, and general practice/service delivery-based education. Hence, other education models are necessary to strengthen the base for public health suicide prevention efforts. A systematic multidisciplinary tertiary education at undergraduate and postgraduate levels can address the multidisciplinary phenomenon of suicide and the prevention approaches required [48]. 

Only few tertiary curriculum-based education courses have been developed, mostly in the U.S., and their evaluations have provided positive outcomes [21,24,48]. For instance, social work students taking a comprehensive course on suicide within a Master of Social Work Program showed increases in knowledge, confidence, and preparedness in working with suicidal clients [21]. Similarly, students across a variety of other health disciplines, such as counseling and public health, among others, have reported positive gains in knowledge, perceived clinical care skills, and perceived ability to help self-harming patients [20].

### 2.3. Suicidology Tertiary Education in Australia

To support high-quality suicide prevention in Australia, the Australian Institute for Suicide Research and Prevention (AISRAP), Griffith University, Queensland, in 2001 commenced teaching two world-first postgraduate programs in suicidology—the Graduate Certificate in Suicidology and the Master of Suicidology. The author D.D.L. first conceived the program in 2000 and, together with J.H., designed and developed the curriculum informed by science and industry needs. From 2004, these programs were offered in both online and in-person (on-campus) mode, and from 2008 were offered in fully online mode to respond to demands of access by interstate and international students, removing barriers and obstacles such as time and geographical location (regarding on-campus attendance) (see [49,50] for an overview of the current programs). These programs aim to equip graduates with the knowledge, attitudes, and skills necessary to utilize theory, research, and clinical principles to develop and implement suicide prevention policy and research and to respond to suicidal persons and people bereaved by suicide. These programs target a diverse range of clinical and nonclinical workers, as well as policymakers and research and academic student populations who may require a deeper education than that provided by other types of training. In particular, graduates of these programs gain advanced knowledge in the field of suicidology, informed by the latest international research in suicide prevention, intervention, and postvention from diverse multidisciplinary perspectives. Furthermore, since 2017 AISRAP’s curriculum has increasingly included lived experience perspectives being integrated into contemporary practice and theory related domains [51].

#### Tertiary Education: Content and Delivery Modes in a Changing Learning Environment

In the two decades since AISRAP’s suicidology programs were introduced, technology, population lifestyles, and the Australian suicide prevention landscape have undergone substantial changes. While these programs have been continually updated over their lifetime to ensure they are informed by the latest Australian and international research, a major curriculum review, informed by perspectives from the suicide prevention sector (lived and living experiences of people touched directly and indirectly by suicide, suicide prevention services, policy, and research), has not been conducted. In addition to sector-related needs around workforce capabilities, there has been a major and rapid change in tertiary education learning. This change has been driven by access to fast-paced technology and online learning platforms, and the changed lifestyle of the work/family life balance is where part-time study and modulized learning are increasingly demanded [52,53]. Additionally, in response to the recent COVID-19 pandemic, along with universities globally, AISRAP’s suicidology programs have been challenged with an urgent need to further enhance accessibility and rapid sophistication in web-based functionality [54]. 

In order to ensure that the programs are meeting the current need of the suicide prevention sector, we conducted a mapping of sector needs against existing curriculum, a form of process evaluation [55]. We planned to explore contemporary suicide prevention sector perspectives on: (a) The importance of a range of suicidology-specific knowledge and skill areas, and (b) preferences on modes of delivery for suicidology education. The results will inform updates of AISRAP’s programs to ensure that they continue to meet the contemporary needs of the sector and to upskill them accordingly. The updates include the introduction of lived experiences into the curriculum, enhanced engagement in online platforms, and trialing “micro/modulized” professional development style offerings to test out the uptake. It is critical that course and program design involves ongoing review to ensure student quality learning in line with sector/industry priorities and emerging technologies and environmental change.

## 3. Materials and Methods

### 3.1. Participants and Recruitment

We conducted an online stakeholder survey in 2018. All participants were required to provide informed consent, and the study was approved by Griffith University’s human research ethics committee (GU Ref: 2017/803). The survey was disseminated through AISRAP’s national email database of stakeholders (national and state-based) engaged in suicide prevention and mental health services (approximately 149 stakeholders). In total, 76 stakeholders participated from different organizations, including governmental and nongovernmental organizations, tertiary and other educations settings, suicidology-specific settings, educational settings, and Aboriginal and Torres Strait Islander-specific organizations. Students or graduates of the suicidology programs were not included.

### 3.2. Questionnaire

The current curriculum learning outcomes from Griffith University’s postgraduate suicidology programs guided the development of the questionnaire. The questionnaire listed 10 suicidology-specific knowledge and skill areas that were taken from the existing learning outcome domains and key competencies of the Graduate Certificate in Suicidology and the Master of Suicidology programs. Stakeholder participants rated each item on a five-point Likert-type scale from “not at all important” to “very important.” Further questions requested information on the type of qualifications considered to be relevant for suicide prevention (postgraduate training, advanced research training, short skills-based course, and others), preferred delivery mode (online modulized with a choice of one or more modules of interest, online courses leading to a degree qualification, and others), the type of professional development they would be willing to support as an employer (formal professional development, informal professional development, and others), and the type of support they would be willing to provide to an employee to engage in this education (financial, study leave, and others).

### 3.3. Analyses

Descriptive analyses were performed using simple frequencies.

## 4. Results

### 4.1. Importance of Key Learning Domains

**Figure 1:**
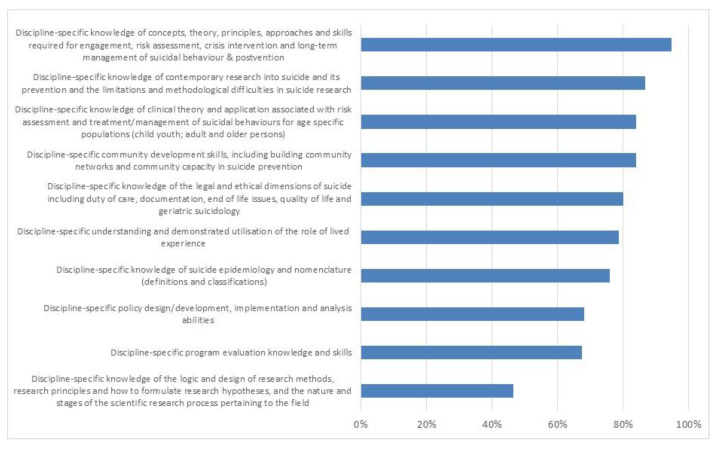
Perceived importance of the key learning domains.

Nine out of the 10 key learning domains were rated by at least 67.4% of the stakeholders as ”important” or “very important” (Figure 1). The four learning domains that were rated the highest were “risk assessment and management” (94.7% of stakeholders rated it either as “important” or “very important”), “contemporary research” (86.7%), “risk assessment and management for age-specific populations,” and “community development skills” (both 84.0%). Furthermore, most stakeholders (78.7%) rated “lived experiences” as either “important” or “very important.” “Logic and design of research methods” was the learning domain that was the least valued by the stakeholders (46.6% of the stakeholders; Figure 1).

### 4.2. Relevant Qualifications and Preferred Mode of Delivery 

According to most of the stakeholders, skills-based qualifications were the most relevant type of qualification (71.1%), followed by postgraduate training (60.5%), qualifications in advanced research training (26.3%), and others (23.7%), such as experiential learning, workshops, and accreditations/degrees. The stakeholders preferred online modulized education (35.5%) with a choice of one or more specialized modules of interest to the work role. This was followed by a combination of both online modulized education and online courses, leading to a degree qualification (34.2%), an online degree qualification only (14.5%) and face-to-face learning (10.5%).

### 4.3. Stakeholder Preference and Willingness to Support Professional Development 

The most favored choice (65.8%) of professional development was a combination of formal professional development, such as structured workshops, seminars, and conferences, and informal professional development, such as supervision and peer mentoring. This was followed by formal professional development alone (22.7%), informal professional development alone (10.5%), and others (11.8%), such as a combination of lived experience and professional development (e.g., telehealth webinars and awareness webinars), and peer accreditation. Half of the stakeholders (50.7%) were willing to support the suicide prevention professional development of their employees by a combination of financial support and study leave. Others were willing to provide study leave (24.0%) or financial support (6.7%). Furthermore, almost one in five of the stakeholders (18.7%) indicated that they would be willing to provide some other form of support, such as supervision and mentoring.

## 5. Discussion

Tertiary-based suicidology education is an important form of deep, comprehensive, and specialized learning in suicide prevention, to be differentiated from gatekeeper and other specialized trainings focused primarily on clinical competencies [19,20]. Griffith University, in Queensland, Australia, has been delivering postgraduate suicidology programs since 2001. Continual improvement and mapping of the evidence-based suicidology curriculum with suicide prevention sector needs was deemed critical for ensuring that student learning outcomes remain well aligned with the knowledge, skills, and attitudes required of them in a fast-changing environment. This form of mapping of needs against existing curriculum is an important process evaluation that can inform future program development, as opposed to the traditional needs assessment processes that inform the original development of programs [55]. Our online survey with sector stakeholders to determine the priority curriculum domains and delivery modes revealed that more than half of the stakeholders deemed nine out of the 10 key learning domains currently embedded in AISRAP’s suicidology programs either as important or very important. This finding indicates that AISRAP programs overwhelmingly meet the expectations and needs of suicide prevention stakeholders in Australia. The stakeholders were interested in tertiary education on the concepts, theory, principles, approaches, and skills required for engagement, risk assessment processes and application, crisis intervention, and long-term management of suicidal behavior and postvention, as well as the clinical theory and treatment/management of suicidal behaviors for age-specific populations (children, youth, adults, and older persons). This can reflect the interest of the suicide prevention sector in the translation of knowledge to application or “hands on” suicide prevention. This conclusion is supported by the stakeholders’ preference of skills-based qualifications as the most relevant type of qualification in suicide prevention. Simultaneously, the survey revealed the need for a solid evidence base of suicide prevention activities, as demonstrated by appreciation and high scores given to “knowledge of contemporary research into suicide and its prevention and the limitations and methodological difficulties in suicide research”.

The stakeholders underlined the need for knowledge on community development skills, including building community networks and community capacity in suicide prevention. This may indicate the interagency characteristic of suicide prevention and networking in Australia, as well as interest in developing community-based initiatives and programs to meet local needs. This is in line with the recent shifts at the national policy level in suicide prevention in Australia, which includes a move to regional administration and the direction of suicide prevention, in the form of Primary Health Network and Local Health District integrated regional planning for mental health and suicide prevention, Department of Health and Human Services-based initiatives [56]. This more localized approach to suicide prevention was in response to the need for more tailored and contextualized approaches that might yield more effective outcomes for communities; for instance, through acknowledging diversity and differences in rural versus urban suicide rates [57]. 

Almost eight out of the 10 stakeholders recognized the importance of discipline-specific understanding and demonstrated utilization of the role of lived experience in design, delivery, implementation, and evaluation of suicide prevention initiatives. This reflects the sentiment of the “Compassion First” report developed by the National Suicide Prevention Taskforce as part of the Interim Advice to the Prime Minister: “Australia’s approach to suicide prevention must be informed by the experiences and wisdom of people with lived experience of suicide and recovery” [58]. Furthermore, this finding is in line with the increasing involvement of people with lived experiences or “consumers” in the development and delivery of services [59] and education programs for health professionals [51,60]. A coproduction approach with people with lived experiences of suicidality creates richer learning outcomes for the student and the person/s and/or populations that they may be supporting [61,62]. Of note, the AISRAP tertiary curriculum includes lived experiences via the suicidology programs’ workforce advisory committee and integration of lived experience into the teaching modules, including short video recordings and relevant literature. 

Given the stakeholders’ interest in skill-based applications of knowledge, it is not surprising that specific knowledge of the logic and design of research methods, research principles, and the nature and stages of the scientific research process pertaining to the field of suicidology was deemed the least important key learning domain. This finding may reflect the priorities of stakeholders who may expect practical benefits (e.g., skills-based) if they are predominantly “service-based” stakeholders (majority of our sample). It may also imply that stakeholders conceive students in suicidology as practitioners, rather than researchers. The finding is important as it reveals a challenge to the program providers to advocate the crucial role of “translation”, and to educate the suicide prevention sector about the importance of research for informing implementation of practice and services and writing and implementing government policy. 

At the same time, it should be stressed that a quarter of stakeholders indicated an interest in advanced research training qualifications. This may reflect a need to access research-specific education in suicidology in Australia, which should be available in parallel to the applied aspects of the study of suicide and its prevention. Consistent with the emerging quality assurance and quality competency frameworks for workplaces and workforces [28,63], this demonstrates the significance of enhancing stakeholder understanding of the importance of evidence-informed approaches.

Most stakeholders were interested in a wide range of professional development formats, including both formal and informal professional development and ranging from conferences, structured workshops, and seminars to supervision and peer mentoring. This reflects the diversity of educational needs of suicide prevention workforce in Australia and the wide range of settings, including clinical work with people at risk. Reflecting the general move to online technology in delivery of education and training [52,53], most stakeholders expressed the need for online education. Nonetheless, there is also an interest in face-to-face education, which should not be overlooked. This observation is supported by the literature, which shows the importance of interactive and blended (online and in-person) education in suicide prevention, which can address different learning styles [20,64,65]. 

It is encouraging that the stakeholders expressed their interest in supporting the workforce needs in suicide prevention training, including financial support and/or study leave. These considerations around postgraduate curriculum are of particular relevance in relation to the current discussion around the certification of suicidologists [66]. Suicidology, or the study of suicide and its prevention, has been originally defined as an “interdisciplinary profession, involving individuals with a wide variety of backgrounds in science, medicine, health, and allied fields” [67] (p.1). Berman et al. [66] proposed that, at a minimum, the core knowledge set of suicidology could include a basic understanding of the epidemiology of suicide and access to current data, suicide risk assessment and treatment, evidence-based knowledge of risk and protective factors for suicide, as well as prevention, intervention, and postvention strategies. Furthermore, lived experiences can be invaluable in enhancing suicidology knowledge and should be integrated in a reciprocal way into the development and design of tertiary suicidology [58].

There is an ongoing need for the standardization in suicide prevention education and training in Australia and internationally [68,69]. For instance, Cramer et al. [19] proposed 10 evidence-based core competencies in regard to suicide risk assessment to inform the development of clinical and counseling psychology programs. Similarly, Hawgood et al. [28] presented four minimum competencies, which are important for the design, delivery, and evaluation of gatekeeper suicide prevention training programs. As of now, there are no standardized competencies in tertiary suicide prevention education, which is a significant gap to be redressed. Due to its vast experience in suicidology curriculum-based tertiary education, AISRAP is well placed to contribute to developments in this field.

### 5.1. Limitations

Our survey had some limitations. It relied on ratings from self-selected stakeholders. It is not known how representative the participating stakeholders are for the field of suicide prevention in Australia, nor if they represent the opinions of potential “students” in suicidology. Nonetheless, we collected the views of a substantial number of stakeholders interested in tertiary suicide prevention education with a reasonably high response rate (51%).

### 5.2. Implications 

The stakeholder survey provided invaluable feedback regarding stakeholders’ views and priorities for such education, which will inform the future development of AISRAP’s suicidology programs. This will ensure that evidence-based education programs are provided that offer a deeper understanding of the phenomenon of suicide and its prevention and provide foundational knowledge to support enhanced skills that students can apply in their respective roles and settings. 

Stakeholders’ high interest in skills and practice-based education in the form of engagement, risk assessment, intervention, and management of suicidality, as well as postvention, should be seriously considered by key university curriculum decision-making bodies when developing or enhancing suicide prevention-related course content. Additionally, the inclusion of education on community capacity, networking, and community collaboration in suicide prevention should be considered, especially in the context of suicide prevention sector reform focusing on integrated regional planning and localized approaches. Finally, the emphasis on need for increased lived experience participation in suicidology education design and delivery is critical. Major curriculum changes are considered appropriate when aligned with industry needs, and efforts to support these changes are limited only by resource allocation limitations. Given the widespread mental health distress and adversity being experienced, particularly by young people [70], since the beginning of the COVID-19 pandemic, it is recommended that these curriculum changes be given serious, immediate consideration for inclusion in both suicidology-specific education programs and related mental health disciplines, such as psychology. 

The finding that research knowledge was rated as the least important education topic suggests a lack of understanding about how research influences evidence-based suicide prevention. Further integration of research and practice-based learnings, as well as increasing the emphasis on translation and implementation research in the courses, should go some way to addressing this finding. 

Overall, the high proportion of stakeholders who rated AISRAP’s existing learning domains as either important or very important provides further support for curriculum-based tertiary education as a valuable component of suicide prevention education. However, the available literature shows a scarcity of curriculum-based tertiary suicidology education options, both in Australia and internationally. To address this, university curriculum decision-making bodies should give priority consideration to the creation of additional suicidology education programs. The finding that many employers in the suicide prevention sector are willing to provide both financial assistance and study leave supports the potential viability of additional suicidology education programs, at least in the Australian context.

## 6. Conclusions

Four key learning domains of tertiary suicide prevention curricula were rated as the most important by stakeholders of the suicide prevention sector in Australia. Skills-based qualifications were the most relevant type of qualification, and online modulized education was the most preferred delivery mode. Half of stakeholders were willing to support suicide prevention professional development through a combination of financial support and study leave.

## Data Availability

Data can be requested from the corresponding author.
